# Performance evaluation of Nile tilapia (*Oreochromis niloticus*) fed diets supplemented with *Lactobacillus plantarum*, *Bacillus subtilis* and *Candida infanticola*

**DOI:** 10.1007/s42770-026-02112-6

**Published:** 2026-10-09

**Authors:** Wanderson Duarte Penido, Diego Fernandes Livio, Vinícius Souza Tarabal, Luísa Ferreira da Cruz, Alessandra de Almeida Rosa Penido, Gabriela Celeste Moreira da Silveira, Layla Conceição Aparecida dos Santos, Dayvid Vincent de Araújo Dutra, Michelle Lucia Diniz Melo, Danilo José Santos Gomes, Newton Moreno Sanches, Ralph Gruppi Thome, Thaís Paula de Araújo, Eloisa Simões Saliba, Paulo Afonso Granjeiro, Juliana Teixeira Magalhães, Carlos Henrique Figueiredo Vasconcellos, Daniel Bonoto Gonçalves

**Affiliations:** 1 Centro-Oeste Campus, Federal University of São João del-Rei, Divinópolis, MG Brazil; 2 Pará de Minas Catholic College, Pará de Minas, MG Brazil; 3 Florestal Campus, Federal University of Viçosa, Florestal, MG Brazil; 4 Department of Animal Science, Federal University of Minas Gerais, Belo Horizonte, MG Brazil; 5 Department of Biotechnology, Federal University of São João del-Rei (UFSJ), Dom Helvécio Square, 74, São João del-Rei, MG 36301-160 Brazil

**Keywords:** *Bacillus subtilis*, Probiotics, Pisciculture, Nile Tilapia, Pathogens

## Abstract

Aquaculture is the fastest-growing agricultural activity globally, providing healthy food with comparatively low environmental impact. However, high stocking densities can generate excessive organic matter, causing stress and increasing pathogen susceptibility in fish. This study evaluated the effects of dietary supplementation with *Candida infanticola* UFSJ7A, *Bacillus subtilis* ATCC 19,659, and *Lactobacillus plantarum* UFSJP2 on Nile tilapia (*Oreochromis niloticus*) performance, intestinal morphology, in vitro antagonistic activity against fish pathogens, and pond water quality. Supplementation with *B. subtilis* significantly improved feed conversion ratio, showing results similar to those of *L. plantarum* and microbial consortium treatments. Dietary supplementation significantly affected feed intake and feed conversion ratio. Fish fed *L. plantarum* showed the lowest feed intake, whereas the microbial consortium resulted in the highest feed intake. Supplementation with *B. subtilis* improved feed conversion ratio, with values comparable to those observed for *L. plantarum* and the microbial consortium. No significant differences were observed in final weight, weight gain, or survival among treatments. No significant effects were observed on fish viability, with survival rates above 90% across treatments. Exploratory intestinal morphometric observations indicated greater microvillus height in fish supplemented with *L. plantarum* and *B. subtilis*, suggesting increased nutrient absorption capacity. In vitro antagonism assays demonstrated that only *B. subtilis* inhibited the growth of *(A) hydrophila*, whereas *L. plantarum* and *C. infanticola* exhibited no antagonistic activity against the tested pathogens. None of the dietary treatments negatively affected water quality, which remained within suitable ranges throughout the experimental period. Overall, dietary supplementation with *(B) subtilis* improved feed utilization, whereas *B. subtilis* and *L. plantarum* were associated with exploratory changes in intestinal morphology. Moreover, the in vitro antagonistic activity exhibited by *B. subtilis* highlights its potential for future investigations on disease prevention strategies in Nile tilapia.

## Introduction

Brazil is among the largest fish producers globally, ranking 4th among the leading producers of tilapia (*Oreochromis niloticus*) [1]. Among farmed fish species, Nile tilapia is particularly prominent commercially due to its rapid growth, tolerance to extreme environmental conditions, high resistance to stress and diseases, and ability to reproduce in captivity. These characteristics have positioned tilapia as an ideal candidate for aquaculture worldwide [2].

Intensive fish farming systems utilize high fish densities along with large quantities of high-protein diets to enhance productivity. However, elevated stocking densities combined with diets rich in nitrogen negatively affect water quality, leading to increased fish stress and reduced immunity [3, 4]. Disease outbreaks under these conditions are particularly concerning because they may cause lesions, impair fish health, increase mortality, and ultimately result in substantial economic losses to producers [5]. Among bacterial diseases, the microbial agents responsible for the most substantial economic impacts on commercial production include *Edwardsella* spp., *Flavobacterium columnare*, *Aeromonas* spp., *Francisella* spp., and *Streptococcus* spp [5].

Several technologies have been employed in fish farming to promote animal welfare. Among these technologies, probiotics stand out. Probiotics are live microorganisms introduced into the gastrointestinal tract via food or water, offering health benefits by enhancing internal microbial balance [6]. Their use in aquaculture presents numerous advantages, such as preventing disease outbreaks, improving feed conversion ratio efficiency, stimulating growth in farmed fish, promoting antioxidant activity, supporting healthy intestinal microbiota proliferation, enhancing the production of digestive enzymes (including protease, amylase, and cellulase), strengthening immunity, reducing stress vulnerability, and improving water quality [7]. Guimarães et al. (2020) isolated and evaluated the probiotic capacity of wild strains of *Lactobacillus plantarum* originating from the biodiversity found in an artisanal cheese produced in Minas Gerais [8]. Among these *L. plantarum* strains, UFSJP2 is the focus of the present study.

Another technology employed in fish farming is the use of biosurfactants. These active molecules enhance the emulsification and solubilization of hydrophobic compounds and have attracted considerable interest because of their broad spectrum of biological activities [9]. Biosurfactants have been widely investigated in the pharmaceutical, food, cosmetic, textile, petroleum, and agricultural industries because of their antimicrobial, antifungal, antiviral, and antibiofilm properties, as well as their therapeutic and biomedical potential [10]. In a previous study conducted by our research group, *Candida infanticola* UFSJ7A was isolated from textile effluent and characterized as a biosurfactant-producing yeast capable of significantly reducing the surface tension of water [11]. Although this strain has not previously been evaluated as a probiotic in aquaculture, its biosurfactant-producing capacity and the recognized antimicrobial properties associated with microbial biosurfactants motivated its selection as an experimental dietary supplement. Thus, the present study investigated the potential application of this strain in Nile tilapia rather than assuming previously established probiotic properties.

Although several studies have reported beneficial effects of probiotic supplementation in Nile tilapia, the beneficial effects of probiotics are highly strain-dependent, and little information is available regarding the use of biosurfactant-producing microorganisms, particularly *Candida infanticola*, as dietary supplements in aquaculture. Furthermore, the combined application of these microorganisms as a microbial consortium has been poorly explored. Therefore, evaluating these specific microbial strains individually and in combination may provide new insights into their potential application in Nile tilapia farming.

In this context, the present study aimed to evaluate the effects of dietary supplementation with the biosurfactant-producing yeast *Candida infanticola* UFSJ7A, the biosurfactant-producing bacterium *Bacillus subtilis* ATCC 19,659, the lactic acid bacterium *Lactobacillus plantarum* UFSJP2, and their microbial consortium on the growth performance, survival, intestinal morphohistology, in vitro antagonistic activity against fish pathogens (*Aeromonas hydrophila*, *Francisella orientalis*, and *Streptococcus agalactiae*), and water quality of Nile tilapia (*Oreochromis niloticus*) fingerlings.

## Materials and methods

### Microorganisms

The bacterial strains *L. plantarum* UFSJP2 and *B. subtilis* ATCC 19,659, together with the yeast *C. infanticola* UFSJ7A, were used as dietary supplements. The pathogenic bacteria *S. agalactiae*, *A. hydrophila*, and *F. orientalis* were used (in vitro) for antagonism analysis.

Microorganism strains: *L. plantarum* UFSJP2 was activated in MRS medium prepared with supplemented whey and incubated at 37 °C for 18 h, as described previously [12]. *B. subtilis* ATCC 19,659 was activated in Nutrient broth, cultivated in an optimized medium composed of soybean flour extract (50 g/L), crystal sugar (20 g/L), and L-arginine (1 g/L), and incubated at 37 °C for 24 h under agitation at 200 rpm, according to the methodology described previously [13]. *C. infanticola* UFSJ7A was activated in Sabouraud broth supplemented with ampicillin (1 µL/mL) and subsequently cultured in supplemented medium containing glucose (25 g/L), sodium chloride (0.10 g/L), calcium chloride (0.10 g/L), ammonium sulfate (2 g/L), magnesium sulfate (0.2 g/L), monopotassium phosphate (0.2 g/L), and yeast extract (5 g/L). Cultures were incubated at 28 °C for 24 h under agitation at 180 rpm, following the method described previously with modifications [14]. After cultivation, microorganisms were sedimented by centrifugation, lyophilized, and then evaluated for cell viability.

*S. agalactiae* ATCC 13,813 was cultured in BHI broth at 30 °C for 24 h under agitation at 200 rpm [15]. *A. hydrophila* IOC/FDA 110 − 36 was cultured in BHI broth at 30 °C for 24 h [16]. *F. orientalis* was grown in Mueller Hinton broth supplemented with 1% glucose and 0.1% L-cysteine, incubated at 28 °C for 48 h [17]. Following cultivation, these microorganisms were centrifuged, lyophilized, and subsequently assessed for cell viability.

### Viability of microorganisms in feed

The viability analysis of microorganisms in the feed was performed as follows: colonies of *L. plantarum* UFSJP2, *B. subtilis* ATCC 19,659, or *C. infanticola* UFSJ7A grown for 24 h were suspended in sterile saline solution to achieve turbidity equivalent to 0.5 McFarland units (approximately 1.5 × 10^8^ CFU/mL). Subsequently, 2 mL of each microorganism suspension were inoculated into 10 g portions of feed previously autoclaved at 121 °C for 15 min. The material was homogenized and incubated at 37 °C for 24 h. After incubation, 1 g of the sample was suspended in 9 mL of sterile saline solution, and serial dilutions were prepared in sterile saline solution up to a dilution of 10^− 3^. Aliquots (100 µL) of the appropriate dilution were plated onto MRS agar for *L. plantarum*, Nutrient agar for *B. subtilis*, and Sabouraud agar for *C. infanticola*. The plates were incubated under the optimal growth conditions for each microorganism, namely at 37 °C for *L. plantarum* and *B. subtilis* and at 28 °C for *C. infanticola*, after which colony-forming units (CFU) were counted.

The viable counts presented in Table 1 correspond to the number of culturable microorganisms recovered from 1 g of supplemented feed immediately after preparation (day 0) and after 7 days of storage. These values were used exclusively to verify microbial viability during feed storage and should not be interpreted as the initial inoculum concentration used for diet supplementation.

**Table 1:** Viable microbial counts recovered from supplemented feed immediately after preparation (day 0) and after 7 days of storage

Microorganisms	Viability in feed mean (± SD)	Viability in feed mean (± SD)
	0 days	7 days
*L. plantarum*	6 × 10^5^ (± 2.30 × 10^5^)	4.6 × 10^5^ (± 1.31 × 10^5^)
*B. subtilis*	6.8 × 10^5^ (± 3.05 × 10^5^)	7.2 × 10^5^ (± 1.23 × 10^5^)
*C. infanticola*	3.6 × 10^4^ (± 1.08 × 10^4^)	4.9 × 10^4^ (± 1.50 × 10^4^)

Results are expressed as colony-forming units (CFU) recovered from 1 g of feed (mean ± SD)

### Performance evaluation of *O. niloticus* supplemented with *L. plantarum*, *B. subtilis* and *C. infanticola*

Three hundred Nile tilapia fingerlings, weighing approximately 14 g (± 0.5) and with an average length of approximately 7.5 cm (± 1.0), were distributed in a completely randomized design comprising five treatments, six replicates, and ten fish per experimental unit. The fish were maintained in 30 plastic tanks (0.1 m³ each) connected to a recirculating aquaculture system (RAS) for the duration of the experiment. Freshwater supplied from a lagoon, was continuously pumped through a physical filter followed by a biological filtration unit and redistributed to all experimental tanks, providing approximately one complete water renewal per hour (0.1 m³ h⁻¹) in each experimental unit. Each tank was equipped with an individual thermostat-controlled heater and independent aeration provided by a porous air stone connected to a central radial blower. Tanks were siphoned daily to remove feces and uneaten feed, and fish were maintained under a 12 h light:12 h dark photoperiod throughout the 31-day experimental period.

The animals were fed twice daily (07:30 h and 15:30 h) until apparent satiety, receiving diets with or without supplementation of *L. plantarum* UFSJP2, *B. subtilis* ATCC 19,659, and *C. infanticola* UFSJ7A. After each feeding, any feed remaining on the water surface was carefully collected before tank cleaning, stored separately for each experimental unit at − 20 °C, and retained for subsequent determination of feed intake. Five dietary treatments were evaluated: (1) *L. plantarum* (1 × 10⁸ CFU/400 g feed); (2) *B. subtilis* (1 × 10⁸ CFU/400 g feed); (3) *C. infanticola* (1 × 10⁸ CFU/400 g feed); (4) a microbial consortium consisting of *L. plantarum*, *B. subtilis*, and *C. infanticola*, with each microorganism added at 1 × 10⁸ CFU/400 g feed (total supplementation of 3 × 10⁸ CFU/400 g feed); and (5) the control diet supplemented only with sterile saline solution (0.85%).

At the end of the experimental period, all feed remaining on the water surface that had been collected daily from each experimental unit was thawed, dried in a forced-air oven at 55 °C to constant weight, and weighed. Feed intake was calculated as the difference between the amount of feed offered and the dry weight of the recovered uneaten feed. Fish were then counted, weighed, and measured to evaluate zootechnical variables according to [18].

Each tank was considered the experimental unit for the evaluation of zootechnical performance. Final weight, weight gain, feed intake, feed conversion ratio, and survival were calculated using the mean values obtained from each tank (*n* = 6 independent tanks per treatment) prior to statistical analysis.

### Intestinal morpho-histological analysis

On the day following the end of the tilapia performance evaluation experiment, fish were fasted for 12 h to ensure complete emptying of the digestive tract and subsequently euthanized with an overdose of eugenol (6 mL L⁻¹; eugenol: ethanol solution, 1:10) [19]. Fish were maintained in the anesthetic solution for at least 5 min after complete cessation of opercular movements and the vestibulo-ocular reflex to ensure death before tissue collection [20]. The abdominal cavity was opened, and a 3-cm intestinal segment located approximately 2 cm distal to the stomach was collected and fixed in formalin for morphohistological analysis [21].

Two fish were sampled from each experimental unit (six tanks per treatment), resulting in 12 fish evaluated per treatment. Histological sections prepared from these fish were pooled for morphometric evaluation. From this set of histological sections, 40 well-oriented intestinal villi were randomly selected for each treatment (*N* = 200 villi). These villi were used as the histological structures for sampling, whereas microvillus height was the morphometric parameter evaluated [22]. Microvillus height was measured in micrometers (µm) using Motic Images Plus 2.0 under light microscopy with a 40× objective. For each selected villus, microvillus height was measured in three predefined regions distributed along the villus surface: the basal, middle, and apical regions [23]. Thus, three morphometric measurements were obtained per villus, resulting in 120 measurements per treatment and 600 measurements overall. The microvilli included in the morphometric analysis were randomly selected from the histological sections of the 12 sampled fish, irrespective of the fish or tank of origin, and were used to characterize the intestinal morphology of each dietary treatment.

### In vitro analysis of antagonism of *L. plantarum*, *B. subtilis*, and *C. infanticola* against pathogenic microorganisms

 In vitro antagonism analyses were performed according to the methodologies described previously with modifications [23]. *L. plantarum* culture was activated in MRS broth, incubated without agitation at 37 °C for 24 h. *B. subtilis* culture was activated in Nutrient broth under agitation at 200 rpm at 37 °C for 24 h. *C. infanticola* was activated in Sabouraud broth under agitation at 180 rpm at 28 °C for 24 h. After activation, a streak was made using a sterile swab onto plates containing Sabouraud agar for *C. infanticola*, Nutrient agar for *B. subtilis*, and MRS agar for *L. plantarum*. Plates containing *B. subtilis* and *L. plantarum* were incubated at 37 °C for 24 h, while the plates containing *C. infanticola* were incubated at 28 °C for 48 h.

Small aliquots of suspensions of *S. agalactiae*, *A. hydrophila*, and *F. orientalis* were added to sterile saline solution (0.85%) until achieving turbidity equivalent to 0.5 on the McFarland standard. Subsequently, two serial dilutions were prepared to obtain final concentrations of 10^6^ and 10^7^ CFU/mL.

For *L. plantarum*, *B. subtilis*, and *C. infanticola*, small portions of colonies were similarly added to tubes containing sterile saline solution (0.85%) until turbidity equivalent to 0.5 McFarland units was achieved. Using a sterile swab, the pathogens were spread onto plates containing MRS agar supplemented with 1% glucose and 0.1% L-cysteine. Subsequently, three wells were made on each plate using a 1000 µL pipette tip, into which 50 µL aliquots of *L. plantarum*, *B. subtilis*, or *C. infanticola* were inoculated. Plates were incubated at 37 °C for 24 h. After incubation, the antagonistic potential of *L. plantarum*, *B. subtilis*, and *C. infanticola* against the pathogenic microorganisms was evaluated by measuring the diameter of the inhibition halos formed around each well, expressed in millimeters. The assay was performed in triplicate using three independent agar plates (technical replicates) for each microorganism–pathogen combination. Inhibition halo diameters are presented as mean ± standard deviation (SD).

### Physical-chemical analysis of tank water

The physicochemical analyses of the water were performed according to the procedures described in the Standard Methods for the Examination of Water and Wastewater [24]. Because all experimental units were supplied by the same recirculating aquaculture system (RAS), water quality was monitored using samples collected from the common water reservoir after physical and biological filtration, which supplied all experimental tanks.

Water temperature and pH were measured daily using a digital thermometer and a digital pH meter, respectively. Electrical conductivity was determined using a conductivity meter, and dissolved oxygen was measured using a digital oximeter. Total alkalinity, hardness, ammonia, nitrite, and nitrate concentrations were determined weekly using a commercial water analysis kit (ALFAKIT), following the manufacturer’s instructions. The water used in the system originated from a lagoon and met the quality standards recommended for fish farming. Water quality measurements were used to monitor the environmental conditions of the experimental system throughout the study and were summarized as mean values over the experimental period.

### Statistical analysis

Statistical analyses were performed using R software (version 4.1.3; R Core Team, Vienna, Austria). Prior to analysis, data normality was assessed using the Shapiro–Wilk test and homogeneity of variances was verified using Cochran’s test. One-way analysis of variance (ANOVA) was used to evaluate the effect of dietary treatments, adopting a significance level of 5% (*p* < 0.05). Whenever significant differences were detected, means were compared using the Student–Newman–Keuls (SNK) multiple comparison test.

For zootechnical performance and survival, the experimental unit was the tank (*n* = 6 tanks per treatment, with 10 fish per tank). Feed intake, feed conversion ratio, final weight, weight gain, and survival were calculated for each tank prior to statistical analysis. Water quality variables were monitored descriptively throughout the experimental period because all tanks were connected to the same recirculating aquaculture system (RAS); therefore, no experimental unit was defined and no statistical comparisons among treatments were performed.

Intestinal morphometric data were analyzed by one-way ANOVA using the measurements obtained from the randomly selected intestinal microvilli. These measurements were used to characterize treatment-level intestinal morphology and should be interpreted as morphometric observations rather than independent biological replicates. The in vitro antagonism assays were performed in triplicate using three independent agar plates for each microorganism–pathogen combination. Inhibition halo diameters were measured on each plate, and the results are expressed as mean ± standard deviation (mm). Since inhibition was observed only for *B. subtilis* against *A. hydrophila*, the data are presented descriptively.

## Results and discussion

### Assessment of the viability of microorganisms in feed

The results of the viability analyses of microorganisms in the feed are shown in Table 1. No losses of CFU/g of microorganisms in the feed were observed after 7 days of preparation.

The viable microbial counts recovered from the supplemented feed immediately after preparation (day 0) and after 7 days of storage are presented in Table 1. All three microorganisms remained viable throughout the storage period, with no marked reduction in recoverable CFU, indicating that the feed formulation maintained microbial viability under the storage conditions adopted in this study. This stability ensured that viable microorganisms were continuously supplied to the fish throughout the experimental period, supporting the interpretation of the zootechnical and intestinal responses observed subsequently.

### Performance evaluation of *O. niloticus* supplemented with *L. plantarum*, *B. subtilis*, and *C. infanticola*

The zootechnical performance of Nile tilapia fingerlings is presented in Table 2. No significant differences were observed among the dietary treatments for final weight, weight gain, or survival (*P* > 0.05). Feed intake was significantly affected by dietary supplementation (*P* < 0.05), with fish fed the diet supplemented with *L. plantarum* UFSJP2 showing the lowest feed consumption, whereas the microbial consortium treatment showed the highest feed consumption. Feed conversion ratio also differed significantly among treatments (*P* < 0.05). Fish fed the diet supplemented with *B. subtilis* ATCC 19,659 exhibited the lowest feed conversion ratio. This treatment differed significantly from the *C. infanticola* and control treatments but did not differ from the *L. plantarum* or microbial consortium treatments. Survival did not differ significantly among treatments (*P* > 0.05), with all treatments showing survival rates above 90%.

**Table 2:** Zootechnical performance of *O. niloticus* fingerlings fed diets supplemented with *L. plantarum*, *B. subtilis*, *C. infanticola*, a microbial consortium, or the control diet

Treatment	Initial weight (g)	Final weight (g)	Weight gain (g)	Cons. Ration (g)	Feed conversion ratio	Viability %
*L. plantarum*	14.40	48.47	34.07	38.54^b^	1.14^ab^	95.00
*B. subtilis*	14.64	54.37	39.73	42.48^ab^	1.07^a^	95.00
*C. infanticola*	14.23	50.88	36.65	43.53^ab^	1.19^b^	93.33
Microbial consortium	14.30	53.40	39.10	44.54^a^	1.14^ab^	98.33
Control	14.26	50.87	36.60	42.99^ab^	1.18^b^	98.33
CV%	5.72	7.93	10.19	7.29	5.55	6.59
Significance	ns	ns	ns	*	*	ns

*CV (%) * coefficient of variation, *ns * not significant. Different lowercase letters within a column indicate significant differences among treatments (*P* < 0.05)

Although dietary supplementation did not affect final weight or weight gain during the experimental period, the improvement in feed conversion ratio observed in fish fed the diet supplemented with *B. subtilis* suggests a more efficient utilization of dietary nutrients.

Among the evaluated microorganisms, dietary supplementation with *B. subtilis* ATCC 19,659 resulted in the greatest improvement in feed conversion ratio compared to the control diet, whereas the other probiotic treatments yielded intermediate responses that did not differ statistically from the *B. subtilis* treatment. Similarly, a recent study demonstrated that dietary supplementation with *B. subtilis* significantly improved the feed conversion ratio and growth performance of Nile tilapia fingerlings, reinforcing the potential of this bacterial species to enhance feed utilization in aquaculture [25]. In an experiment carried out by Mohammadi et al. (2020) using a strain of *L. plantarum* in biofloc culture systems and stagnant renewal on the zootechnical performance of Nile tilapia, no significant difference was observed for final weight, specific growth rates, feed conversion ratio and total biomass of the probiotic in the different culture systems among the treatments used for a period of 90 days [26]. Guimarães et al. (2019) also did not observe significant differences in the zootechnical performance parameters of growth and survival in their 28-day experiment using *L. plantarum* and *B. subtilis* in Tilapia fingerlings [27].

The absence of significant differences in final weight and weight gain despite improvements in feed conversion ratio highlights that probiotic effects are often strain-dependent and do not necessarily result in immediate growth responses. Differences among studies may be explained by variations in probiotic strains, supplementation period, dietary composition, fish size, rearing conditions, and microbial colonization efficiency. Therefore, the present findings reinforce that improvements in feed efficiency may occur independently of changes in growth performance during relatively short experimental periods.

Elsisy et al. (2026) reported that dietary supplementation with *Yucca schidigera* extract and *Bacillus subtilis* significantly improved growth performance and immune response while reducing ammonia excretion in Nile tilapia under culture conditions [28]. Veiga et al. (2020) included *B. subtilis* in different doses for 10 days in surubim and observed zootechnical benefits sufficient to cause an immunomodulatory effect, influencing the hematological parameters in the animals, which increased blood defense. The authors also found an increase in the height and width of the villi, enabling better nutrient absorption [29].

Although the microbial consortium was expected to combine the beneficial characteristics of the individual microorganisms, its performance was not superior to that of *B. subtilis* alone for feed conversion ratio. This finding indicates that, under the experimental conditions adopted in the present study, combining the three microorganisms did not result in an apparent additive effect on zootechnical performance. These results reinforce that the response to probiotic supplementation depends not only on the individual characteristics of each microorganism but also on their interaction when administered simultaneously.

### Intestinal morpho-histological analysis

**Fig. 1:**
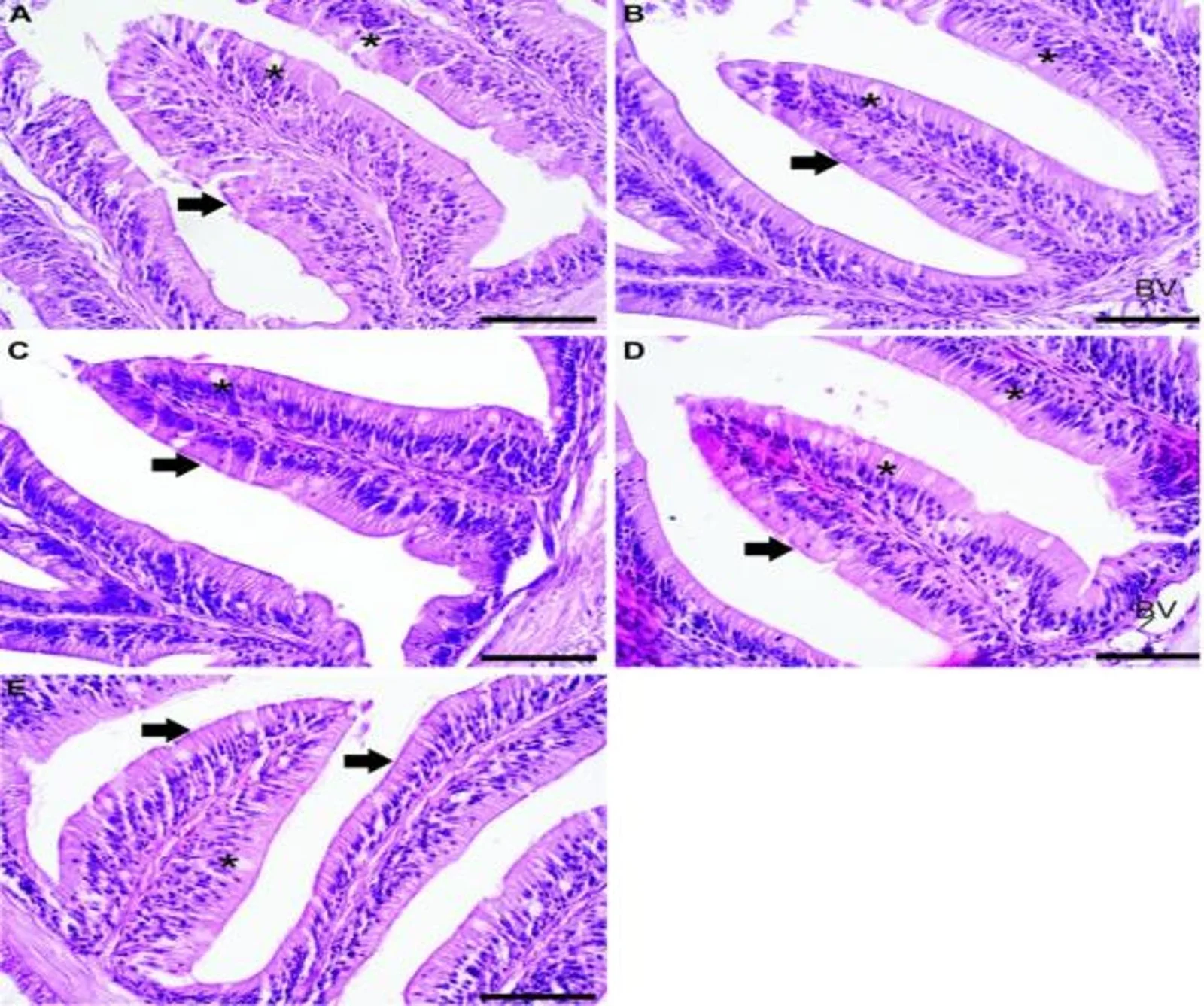
Representative histological images of the intestinal villi of the experimental groups observed under a 40× objective. *L. plantarum* UFSJP2 (**a**), *B. subtilis* ATCC 19,659 (**b**), *C. infanticola* UFSJ7A (**c**), microbial consortium (**d**), and control (**e**). Arrows indicate the intestinal villus regions considered for the morphometric assessment of microvillus height. Asterisks indicate goblet cells. BV indicates blood vessels. Scale bar = 50 μm

Morpho-histological analyses of tilapia for each treatment are shown in Table 3. Fish supplemented with *L. plantarum* UFSJP2 and *B. subtilis* ATCC 19,659 presented the greatest microvillus height values. These exploratory morphometric observations suggest a possible increase in the intestinal absorptive surface area, which may have contributed to improved nutrient absorption, consistent with the improved feed conversion ratio observed in the *B. subtilis* treatment. Figure 1 shows representative images of the intestinal microvilli.

**Table 3:** Intestinal microvillus height of Nile tilapia fingerlings

Group	Microvillus height mean (± SD)
*L. plantarum* UFSJP2	1.736 (± 0.552) µm
*B. subtilis* ATCC 19,659	1.980 (± 0.634) µm
*C. infanticola* UFSJ7A	1.603 (± 0.440) µm
Microbial consortium	1.586 (± 0.332) µm
Control	1.500 (± 0.526) µm
CV %	61.61

Values are presented as descriptive morphometric observations

*CV% * Coefficient of variation

The increase in microvillus height observed in fish supplemented with *L. plantarum* UFSJP2 and *B. subtilis* ATCC 19,659 suggests an improvement in the absorptive capacity of the intestinal mucosa. However, although both microorganisms promoted intestinal morphological changes, only *B. subtilis* resulted in a significant improvement in feed conversion ratio. This finding indicates that improvements in intestinal morphology alone may not always translate into measurable differences in growth performance, as the overall zootechnical response is influenced by multiple physiological and nutritional factors.

The increase in intestinal microvilli observed in the present study is consistent with recent findings reported by Eissa et al. (2024) [30], who demonstrated that dietary supplementation with *Bacillus subtilis* improved intestinal histological characteristics, including villus development and absorptive surface areas, in red hybrid tilapia (*Oreochromis mossambicus* × *O. niloticus*). Similar results have also been reported by Cao et al. (2019) evaluated the effects of supplementing Pengze crucian carp diets with β-glucan and *B. subtilis*, with the aim of evaluating the growth, fillet quality, immunological and antioxidant capacity in the fish, and found that supplementation with *B. subtilis* can increase the intestinal absorption capacity due to the significant increase in the height of the intestinal folds and microvilli [31].

Dawood et al. (2019) carried out a study using *L. plantarum* L 137 as a supplement to diets of GIFT tilapia, with the aim of evaluating the growth, activity of digestive enzymes, intestinal morphology and oxidative state of the fish [32]. Supplementation with *L. plantarum* resulted in an increase in the length of the microvilli in the middle and posterior portions of the intestine. The similarity between the present study and the findings of Dawood et al. (2019) reinforces the beneficial effect of *L. plantarum* on intestinal morphology. Nevertheless, the observed response appears to be strain-dependent, since improvements in intestinal structure are not consistently accompanied by proportional gains in zootechnical performance. This highlights the importance of evaluating individual probiotic strains under specific production conditions.

Ntakirutimana et al. (2023) reported that probiotic supplementation enhances intestinal morphology in Nile tilapia by increasing villus height and improving mucosal development, thereby expanding the absorptive surface area available for nutrient uptake. Although these authors assessed villus (fold) height rather than microvillus height, comparable exploratory increases were observed at the microvillus level, consistent with the increased microvillus height observed in the *L. plantarum* and *B. subtilis* treatments in the present study [33].

Although the microbial consortium also contained *L. plantarum* and *B. subtilis*, it did not produce greater intestinal development than the individual supplementation with these microorganisms. Under the conditions of the present study, combining the three microbial strains did not result in an apparent additive effect on intestinal morphology, indicating that the response to probiotic supplementation depends on the specific interaction between the microorganisms included in the diet.

Improvements in intestinal morphology increase the absorptive surface area and contribute to maintaining intestinal epithelial integrity, thereby enhancing digestive efficiency and overall intestinal health [34, 35]. In the present study, this relationship was particularly evident in fish supplemented with *B. subtilis*, in which greater microvillus height coincided with improved feed conversion ratio.

### In vitro analysis of antagonism of *L. plantarum*, *B. subtilis* and *C. infanticola* against pathogenic microorganisms

**Fig. 2:**
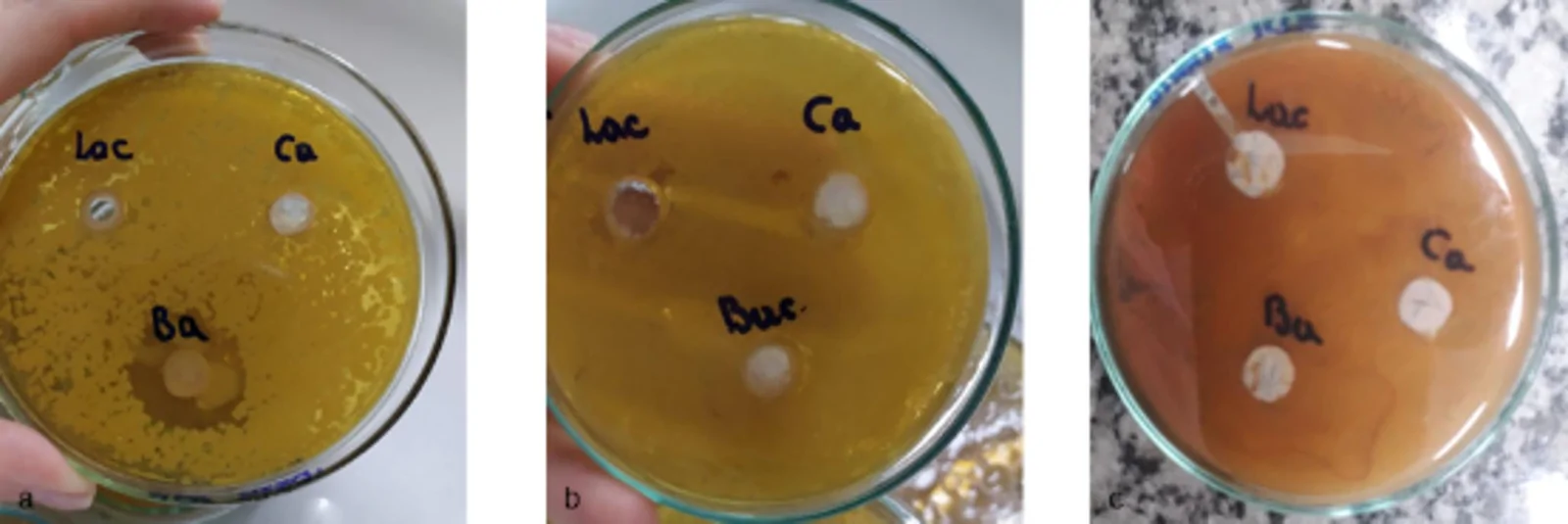
Inhibition zones of microorganisms tested. Inhibition zone of *B. subtilis* in medium containing *A. hydrophila* (a), *F. orientalis* (b), and *S. agalactiae* (c) as pathogen

The results of antimicrobial activity of *L. plantarum* UFSJP2, *B. subtilis* ATCC 19,659, and *C. infanticola* UFSJ7A against *S. agalactiae*, *F. orientalis* and *(A) hydrophila* are represented in Table 4. In Fig. 2 it is possible to see the inhibition halo of *(B) subtilis* against the pathogens of *(A) hydrophila*. The cell suspensions of *L. plantarum* and *C. infanticola* did not inhibit the growth of any of the tested pathogens. However, the bacterium *(B) subtilis* was able to inhibit the development of the *A. hydrophila* pathogen with the formation of halos around the colony. For the other pathogens, there was no formation of inhibition halos. Although *(C) infanticola* UFSJ7A is known to produce biosurfactants [11], no inhibition halos were observed against any of the tested pathogens under the experimental conditions adopted in this study.

**Table 4:** In vitro antagonistic activity of the tested microorganisms against fish bacterial pathogens, expressed as inhibition halo diameter (mean ± SD, mm; *n* = 3 technical replicates)

Pathogens (UFC/mL)	L. plantarum (mm)	B. subtilis (mm)	C. infanticola (mm)
*A. hydrophila* (10^6^)	0	25 ± 0,58	0
*A. hydrophila* (10^7^)	0	24 ± 1,15	0
*F. orientalis* (10^6^)	0	0	0
*F. orientalis* (10^7^)	0	0	0
*S. agalactiae* (10^6^)	0	0	0
*S. agalactiae* (10^7^)	0	0	0

The absence of inhibition halos for *L. plantarum* UFSJP2 and *C. infanticola* UFSJ7A should not be interpreted as evidence that these microorganisms lack probiotic potential. Antimicrobial activity is a strain-dependent characteristic and may vary according to the culture conditions, growth medium, target pathogen, and production of bioactive metabolites. Therefore, the lack of in vitro inhibition observed in the present study does not exclude the possibility that these microorganisms may exert beneficial effects through other mechanisms, such as modulation of the intestinal microbiota or improvement of intestinal health.

Bonin et al. (2019) performed antagonism analyses in their experiment using the well methodology, which used pathogens of *A. hydrophila* and *S. agalactiae* at a concentration of 1 × 10^8^ CFU/mL, and *L. plantarum* [36]. The strain of *L. plantarum* grown in powdered milk (10%) and MRS + milk (2%) showed formation of halos against both pathogens. Mohamed et al. (2024) reported that *Latiplantibacillus plantarum* exerts probiotic effects in Nile tilapia by improving the intestinal microbial balance and stimulating the immune system, thereby enhancing resistance to bacterial pathogens. According to the authors, these beneficial effects are associated with multiple mechanisms, including the production of antimicrobial metabolites, competitive exclusion of undesirable microorganisms, and immunomodulation [37]. The discrepancy between the present results and those reported by Bonin et al. [36] likely reflects differences among probiotic strains and experimental conditions. Although both studies evaluated *L. plantarum*, antimicrobial activity is known to vary considerably among strains, reinforcing the importance of individually characterizing candidate probiotics before their application in aquaculture.

Cao et al. (2019) performed an experiment using a strain of *Bacillus velezensis* at a concentration of 10^8^ CFU/mL in vitro and in vivo in grass carp, and observed the inhibition of the development of *A. hydrophila* [31]. Macias et al. (2024) demonstrated that selected *Bacillus* spp. exhibited in vitro antagonistic activity against *Aeromonas hydrophila*. These strains also improved growth performance and increased resistance to *(A) hydrophila* infection in Nile tilapia, supporting the potential of *Bacillus* spp. as probiotics for disease control in aquaculture [38]. The selective antagonistic activity observed for *(B) subtilis* against *A. hydrophila*, but not against *F. orientalis* or *S. agalactiae*, indicates that the antimicrobial activity of this strain is not universal and may depend on the susceptibility of the target microorganism. This result reinforces the need to evaluate probiotic strains against specific pathogens of interest rather than assuming broad-spectrum antimicrobial activity.

*B. subtilis* has several mechanisms of pathogen inhibition, such as bacteriocin production, suppression of virulence gene expression, competition for adhesion sites, production of lytic enzymes, production of antibiotics, immunostimulation, competition for nutrients and energy, and production of organic acids [39]. *B. subtilis* produces lipopeptides, which are considered one of the most effective biosurfactants, with therapeutic properties, such as antitumor activity. In addition, *B. subtilis* is a suitable microorganism to produce biosurfactants because it does not present pathogenic effects, and its products can be applied directly in the food and pharmaceutical industries [40].

The inhibition of *(A) hydrophila* observed in the present study may therefore be associated with one or more of these antimicrobial metabolites produced by *(B) subtilis*. However, because the present study evaluated antagonistic activity exclusively under in vitro conditions, these results should be interpreted as evidence of antimicrobial potential rather than direct evidence of enhanced disease resistance in Nile tilapia. Further in vivo pathogen challenge studies are required to determine whether this antagonistic activity translates into protection under farming conditions.

### Physical-chemical analysis of tank water

Physical-chemical characteristics of the water in the recirculating aquaculture system (RAS) are presented in Table 5. Because all experimental units shared the same recirculating water system supplied from a common reservoir, these measurements represent the overall water quality monitored throughout the experimental period rather than treatment-specific values. Therefore, water quality parameters were used to verify the maintenance of adequate environmental conditions during the trial and were not subjected to statistical comparisons among dietary treatments. All monitored parameters remained within the ranges considered suitable for Nile tilapia culture, indicating stable environmental conditions throughout the experiment.

**Table 5:** Mean physicochemical parameters of the water monitored weekly in the common recirculating aquaculture system (RAS) during the experimental period

Sampling week	Electrical conductivity µS/cm	pH	Alkalinity CaCo_3_ (mg/L)	Hardness CaCo_3_ (mg/L)	Total ammonia *N*-NO_3_ (mg/L)	Nitrite mg/L *N*-NO_2_(ppm)	Nitrate *N*-NO_3_ (mg/L)	Dissolved Oxygen (mg/L)	Temperature (°C)
Week 1	38.97	7.36	20	20	0.0	0.00	6.0	7.1	27.4–27.6
Week 2	52.03	6.78	20	20	0.0	0.05	8.0	6.2	27.1–27.2
Week 3	37.98	6.57	20	20	0.1	0.05	12.5	5.9	27.4–27.6
Week 4	51.35	6.5	20	20	0.1	0.025	12.5	5.6	27.4–27.5
Week 5	44.32	6.53	20	20	0.1	0.05	12.5	6.0	27.6–27.8

Values represent descriptive monitoring of the shared water reservoir and were not compared statistically among dietary treatments

The recirculating aquaculture system (RAS) maintained stable physicochemical conditions throughout the experimental period. Water quality was monitored only to verify environmental stability within the shared system and was not used to evaluate treatment effects.

Electrical conductivity remained within the recommended range for freshwater aquaculture, indicating adequate ionic conditions for Nile tilapia culture [41, 42]. Likewise, pH values remained within the recommended range (6.5–9.0), while alkalinity and total hardness remained constant at 20 mg/L CaCO₃ throughout the experimental period, meeting the minimum values recommended for fish production [43–46]. These results demonstrate that the recirculating aquaculture system (RAS) provided stable water quality, minimizing environmental fluctuations that could interfere with tilapia performance.

Nitrogenous compounds also remained within acceptable limits during the experimental period. Total ammonia did not exceed 0.1 mg/L, remaining below concentrations considered safe for Nile tilapia culture [47]. The presence of nitrate, associated with consistently low concentrations of ammonia and nitrite, indicates that nitrification occurred efficiently throughout the experimental period, reflecting the biological conversion of ammonia to nitrite and subsequently to nitrate [48]. Dissolved oxygen remained above 5.0 mg/L, a concentration considered adequate to sustain normal feeding behavior, growth performance, and feed utilization in Nile tilapia [49]. Similarly, water temperature remained stable throughout the experimental period (27.1–27.8 °C), remaining within the optimal range reported for the production of this species [50].

Collectively, these results indicate that environmental conditions remained suitable throughout the experimental period and did not influence the differences observed among treatments. Consequently, the improved feed conversion observed in fish supplemented with *B. subtilis*, together with the exploratory intestinal morphometric findings associated with *B. subtilis* and *L. plantarum*, are unlikely to be explained by variations in water quality, since environmental conditions remained stable throughout the experimental period based on descriptive monitoring of the shared recirculating aquaculture system.

## Conclusion

The supplementation of Nile tilapia diets with *B. subtilis* ATCC 19,659 improved feed conversion ratio, indicating more efficient feed utilization under the conditions evaluated in this study. Although improved feed conversion ratio may represent a potential advantage for commercial aquaculture, no economic analysis was performed, and therefore the economic implications of these findings should be interpreted with caution. Dietary supplementation with *L. plantarum* UFSJP2 and *B. subtilis* ATCC 19,659 was also associated with exploratory increases in intestinal microvillus height, which may have contributed to the improved feed conversion ratio observed for the *B. subtilis* treatment by increasing the absorptive surface available for nutrient uptake. The in vitro antagonism assays demonstrated that *B. subtilis* inhibited the growth of *Aeromonas hydrophila*, whereas *L. plantarum* and *C. infanticola* UFSJ7A showed no inhibitory activity against the pathogens evaluated. Water quality remained within the recommended ranges for Nile tilapia throughout the experimental period, indicating stable environmental conditions during the study. Overall, the results suggest that *L. plantarum* UFSJP2 and *B. subtilis* ATCC 19,659 have potential as dietary supplements for Nile tilapia, particularly due to their positive effects on intestinal morphology and, in the case of *B. subtilis*, feed conversion ratio. However, since no pathogen challenge was performed, the observed in vitro antagonistic activity should not be interpreted as evidence of enhanced disease resistance. Further in vivo challenge studies are required to determine whether the antimicrobial activity observed under laboratory conditions translates into protection against infection under farming conditions.

## Data Availability

The datasets generated during and/or analysed during the current study are available from the corresponding author on reasonable request.
